# ﻿Morphology and multigene phylogeny revealed four new species of *Geastrum* (Geastrales, Basidiomycota) from China

**DOI:** 10.3897/mycokeys.113.139672

**Published:** 2025-01-28

**Authors:** Xin Yang, Yonggao Zhu, Songjing Duan, Xingxing Wu, Changlin Zhao

**Affiliations:** 1 College of Forestry, Southwest Forestry University, Kunming 650224, China Southwest Forestry University Kunming China; 2 Yunnan Forestry Technological College, Kunming 650224, China Yunnan Forestry Technological College Kunming China

**Keywords:** Classification, earthstar fungus, Geastraceae, molecular systematics, taxonomy

## Abstract

In the present study, four new species, *Geastrumartocarpicola*, *G.fibulatum*, *G.sinense* and *G.trachelium* collected from China, are proposed based on a combination of morphological characteristics and molecular evidence. *Geastrumartocarpicola* is characterized by shallowly saccate to deep saccate exoperidium, bubble-shaped to flask shaped basidia, spherical basidiospores. *G.fibulatum* is characterized by shallowly saccate to deep saccate exoperidium, spherical basidiospores, generative hyphae with clamp connections in the mycelium layer. *G.sinense* has arched exoperidium, long stipe expanded basidiomata, and spherical basidiospores. *G.trachelium* has deep saccate exoperidium, flask-shaped basidia, and spherical basidiospores. Sequences of the internal transcribed spacers (ITS), large subunit (nrLSU), the largest subunit of ribosomal polymerase II (RPB1), and subunit 6 of ATP synthase (ATP6) of the nuclear ribosomal DNA (rDNA) markers of the studied samples were generated, and the phylogenetic analyses were performed with maximum likelihood, maximum parsimony and Bayesian inference methods. The results showed that our collection clustered within *Geastrum* but distinctly from the others. Full morphological descriptions, illustrations, and phylogenetic analyses results for the four new species are provided. In addition, *G.sanglinense* is treated as a synonym of *beijingense*.

## ﻿Introduction

Fungi are among the most diverse groups of organisms on the earth and play an indispensable role in ecosystem processes and functioning (Wei and Dai 2004; Hyde 2022; Dong et al. 2024a). Currently, 19 phyla of fungi are accepted as Aphelidiomycota, Ascomycota, Basidiobolomycota, Basidiomycota, Blastocladiomycota, Calcarisporiellomycota, Chytridiomycota, Entomophthoromycota, Entorrhizomycota, Glomeromycota, Kickxellomycota, Monoblepharomycota, Mortierellomycota, Mucoromycota, Neocallimastigomycota, Olpidiomycota, Rozellomycota, Sanchytriomycota, and Zoopagomycota (Wijayawardene et al. 2024). Most Basidiomycota species act as decomposers and mutualists of plants and animals, and play fundamental ecological roles such as driving carbon cycling in forest soils, mediating the mineral nutrition of plants, and alleviating carbon limitations of other soil organisms (Tedersoo et al. 2014; Yuan et al. 2023a). The genus *Geastrum* Pers., widely known as the earthstars, belongs to the family Geastraceae (Basidiomycota) (Sunhede 1989; Zamora et al. 2014). *Geastrum* species have been recorded on all continents except Antarctica, mostly in the forest humus layer, and occasionally on rotten wood, sand, or grassland, in which it is characterized by the exoperidium splitting into rays at maturity (León 1968).

*Geastrum* was first proposed by Persoon (1794) and typified by *G.coronatum* Pers. (Persoon 1794). The Gasteromycete means “stomach fungus” and these fungi produce their spores inside the fruiting body that are enclosed inside an outer covering called peridium, and the *Geastrum* is considered one of the most genera-diverse of gasteroid fungi (Chouhan and Panwar 2021; Zhang et al. 2023b). *Geastrum* is characterized by the stelliform basidiomata, exoperidium with three layers, sessile or stalked endoperidium, and sulcate, plicate, folded or fibrillose peristome, distinctly or indistinctly delimited, sometimes with mycosclereids (Sunhede 1989; Zamora et al. 2014). Both Index Fungorum (http://www.indexfungorum.org; 10 October 2024) and MycoBank database (http://www.MycoBank.org; 10 October 2024) have registered 388 specific and infraspecific names in *Geastrum*, but the actual number of species has been estimated to be around 130 (Finy et al. 2021; Wijayawardene et al. 2022; He et al. 2024). Of them, 36 species have been recorded from China (Wang and Bau 2023; Zhang et al. 2023b; Wu et al. 2024; Yang et al. 2024).

The classification studies on *Geastrum* were carried out in China, in which the morphological characteristics were as the core evidence for species taxonomy (Teng 1963; Tai 1979; Zhou et al. 2007). Phylogenetic relationships among European earthstars were inferred using sequence data from the nuclear ribosomal DNA internal transcribed region (ITS1, 5.8S, and ITS2), nuclear large subunit (nrLSU), and translation elongation factor 1-alpha (*tef*1-α), and the results indicated that the phylogenetic analyses recovered 11 clades including thirty-one morphological species (Jeppson et al. 2013). They proposed a close phylogenetic relationship between *Myriostomacoliforme* (Dicks.) Corda and *Geastrumcoronatum* (Jeppson et al. 2013). Later, on the basis of the phylogenetic analyses of four molecular markers as 5.8S nrDNA, nrLSU, the largest subunit of ribosomal polymerase II (RPB1), and subunit 6 of ATP synthase (ATP6), the research indicated that a new subdivision of the genus *Geastrum* was presented, and the phylogenetic and morphological boundaries among 3 genera *Geastrum*, *Myriostoma* Desv., and *Radiigera* Zeller (Geastraceae) were evaluated (Zamora et al. 2014). The genus *Myriostoma* represented a different phylogenetic lineage within the family Geastraceae which was confirmed as distinct from *Geastrum* (Zamora et al. 2014). Inferred from ITS, LSU nrDNA, RPB1, and ATP6, the phylogenetic reconstructions with 95 samples showed that 5 clades were considered as five subsections and a total of 27 lineages were proposed (Zamora et al. 2015). Multigene phylogenetic analyses involving sequences from ITS, nrLSU, RPB1, ATP6, and tef1-α revealed that *Geastrum* formed a distinct clade and had a close relationship with *G.granulosum* s.l. (Finy et al. 2021). Based on the morphological observation combined with phylogenetic analysis of ITS-nrLSU-RPB1-ATP6, the species *G.yanshanense* C.L. Hou et al. and *G.beijingense* C.L. Hou et al., were introduced from Yanshan Mountains, China (Zhou et al. 2022). Based on morphological observation combined with phylogenetic analysis through ITS+nrLSU, 7 new *Geastrum* species were introduced from China (Wang and Bau 2023). In addition, phylogenetic analyses based on sequences of ITS, nrLSU, and ATP6 regions showed that *G.sanglinense* Y.Q. Wu & Shu R. Wang was sister to *G.yanshanense* and *G.rubellum* P.-A. Moreau & C. Lécuru (Wu et al. 2024). Phylogenetic analyses inferred from ITS+nrLSU dataset indicated that *G.yunnanense* Xin Yang & C.L. Zhao was nested within the *Geastrum*, which was forming a monophyletic lineage and then grouped with *G.velutinum* Morgan, and *G.javanicum* Lév. (Yang et al. 2024).

During investigations on earthstars fungi in China, we found four *Geastrum* species that could not be assigned to any described species. Based on the morphological characteristics and molecular phylogenetic analyses, these species are described as *G.artocarpicola*, *G.fibulatum*, *G.sinense* and *G.trachelium*.

## ﻿Materials and methods

### ﻿Sample collection and examination

The fresh basidiomata of fungi growing on the ground were collected from Guangzhou and Yangjiang in Guangdong Province, Nanjing in Jiangsu Province, Xiameng in Fujiang Province, P.R. China. The samples were photographed using a Jianeng 80D camera (Tokyo, Japan) in situ and fresh macroscopic details, such as the color of the mycelial layer, the pseudoparenchymatous layer, the type of endoperideal body, and the shape of the peristome, were recorded. All the photographs were focus-stacked and merged using Helicon Focus Pro 7.7.5 software. Specimens were dried in an electric food dehydrator at 40 °C (Zhao et al. 2023; Dong et al. 2024a, b), and then sealed and stored in an envelope bag and deposited in the herbarium of the Southwest Forestry University (SWFC), Kunming, Yunnan Province, P.R. China.

### ﻿Morphology

The macromorphological descriptions were based on field notes and photos captured in the field and laboratory and followed the color terminology of Petersen (1996). Micromorphological data were obtained from the dried specimens following observation under a light microscope (Zhao and Wu 2017; Wang et al. 2023, 2024; Dong et al. 2024a). Drawings were made with the aid of a fungus plotter (Zhao et al. 2023). The measurements and drawings were made from slide preparations stained with Cotton Blue (0.1 mg aniline blue dissolved in 60 g pure lactic acid), Melzer’s reagent (3 g potassium iodide, 1 g crystalline iodine, 44 g chloral hydrate, and 40 ml distilled water) and 5% potassium hydroxide. Spore size data, excluded 5% of the measurements from each end of the range, showing them in parentheses. The following abbreviations were used: KOH = 5% potassium hydroxide water solution, CB+ = cyanophilous, CB = cotton clue, CB– = acyanophilous, IKI = Melzer’s reagent, IKI– = both inamyloid and indextrinoid. Q = variation in the L/W ratios between the specimens studied and n = a/b (number of spores (a) measured from a given number (b) of specimens). Q_m_ represented the average Q of basidiospores measured ± standard deviation.

### ﻿DNA extraction, PCR amplification, sequencing, and phylogenetic analyses

The CTAB rapid plant genome extraction kit-DN14 (Aidlab Biotechnologies Co., Ltd, Beijing, China) was used to obtain genomic DNA from the dried fungal specimens according to the manufacturer’s instructions (Zhao and Wu 2017; Zhou et al. 2023; Dong et al. 2024a). Four molecular markers were investigated, i.e., ITS (including 5.8S) and 28S (nrLSU) nrDNA, the largest subunit of RNA polymerase II (RPB1), and subunit 6 of ATP synthase (ATP6). The nuclear ribosomal ITS region was amplified with ITS1F and ITS4 primer pair (White et al. 1990). The nuclear nrLSU region was amplified with the LR0R and LR7 primer pair (Vilgalys and Hester 1990; Rehner and Samuels 1994). The RPB1 region was initially amplified with RPB1-Af and RPB1-Cr (Matheny et al. 2002). For amplifying ATP6, ATP6-1 and ATP6-2 were used (Kretzer and Bruns 1999). The PCR procedure for ITS was as follows: initial denaturation at 94 °C for 3 min, followed by 35 cycles at 94 °C for 60 s, 56 °C for 60 s, and 72 °C for 1 min, and a final extension of 72 °C for 10 min (Yang et al. 2024). The PCR procedure for nrLSU was as follows: initial denaturation at 94 °C for 1 min, followed by 35 cycles at 94 °C for 30 s, 48 °C for 1 min, and 72 °C for 1.5 min, and a final extension of 72 °C for 10 min (Yang et al. 2024). The PCR procedure for RPB1 was 94 °C for 2 min, followed by 10 cycles at 94 °C for 40 s, 60 °C for 40 s and 72 °C for 2 min, then followed by 37 cycles at 94 °C for 45 s, 55 °C for 1.5 min and 72 °C for 2 min, and a final extension of 72 °C for 10 min (Dong et al. 2024a). The PCR procedure for ATP6 was as follows: initial denaturation at 94 °C for 3 min, followed by 35 cycles at 94 °C for 35 s, 55 °C for 50 s, and 72 °C for 45 min, and a final extension of 72 °C for 10 min (Kretzer and Bruns 1999). The PCR products were purified and sequenced at Kunming Tsingke Biological Technology Limited Company (Yunnan Province, China). All of the newly generated sequences were deposited in NCBI GenBank (https://www.ncbi.nlm.nih.gov/genbank/) (Table 1).

**Table 1.** List of species, specimens, and GenBank accession number of sequences used in this study. [New species is shown in bold; * type material].

Species Name	Locality	Sample No.	GenBank Accession No.				References
			ITS	nrLSU	*rpb1*	*atp6*	
*Geastrumarenarium*	Argentina	MA-Fungi 83760	KF988351	KF988471	KF988606	KF988741	Zamora et al. (2014)
*Geastrumargentinum*	Argentina	LPS 48446	KF988352	KF988472	KF988607	KF988742	Zamora et al. (2014)
*Geastrumargentinum*	Argentina	MA-Fungi 82605	KF988353	KF988473	–	KF988743	Zamora et al. (2014)
***Geastrumartocarpicola***	**China**	CLZhao 36079*	**PQ484149**	**PQ481917**		**PQ634876**	**Present study**
***Geastrumartocarpicola***	**China**	**CLZhao 39927**	**PQ484150**	**PQ481918**		**PQ634877**	**Present study**
*Geastrumaustrominimum*	Australia	MEL:2276089	KP687490	KP687451	KP687532	KP687573	Zamora et al. (2015)
*Geastrumaustrominimum*	Australia	MEL:2292062	KP687491	KP687452	KP687533	KP687574	Zamora et al. (2015)
*Geastrumbeijingense*	China	BJTC 248	MZ508872	MZ509376	MZ571167	MZ571178	Zhou et al. (2022)
*Geastrumbeijingense*	China	BJTC 073	MZ508873	MZ509377	MZ571168	MZ571179	Zhou et al. (2022)
*Geastrumbeijingense*	China	BJTC 369	–	MZ509375	MZ571166	MZ571177	Zhou et al. (2022)
*Geastrumbeijingense*	China	BJTC 030	MZ508874	MZ509378	MZ571169	MZ571180	Zhou et al. (2022)
*Geastrumbeijingense*	China	BJTC 1523	MZ508875	MZ509379	MZ571170	MZ571181	Zhou et al. (2022)
*Geastrumbenitoi*	Spain	MA-Fungi 68191	KF988350	KF988469	KF988604	KF988739	Zamora et al. (2015)
*Geastrumbenitoi*	Spain	MA-Fungi 87324	KP687494	KP687455	KP687536	KP687577	Zamora et al. (2015)
*Geastrumberkeleyi*	Sweden	Herb.Zamora 504	KF988356	KF988476	KF988611	KF988746	Zamora et al. (2015)
*Geastrumbrunneocapillatum*	Brazil	UFRN:Fungos:2286	MH634996	MH635029	–	–	Accioly et al. (2019)
*Geastrumcampestre*	Spain	Herb. Zamora 283	JN943167	JN939575	JN991286	KF988748	Zamora et al. (2014)
*Geastrumcampestre*	Sweden	Herb. Sunhede 7575	KF988357	KF988479	KF988614	KF988749	Zamora et al. (2014)
*Geastrumcorollinum*	Australia	MA-Fungi 5746	KF988359	KF988481	KF988616	KF988751	Zamora et al. (2014)
*Geastrumcorollinum*	Sweden	Herb. Sunhede 7744	KF988360	KF988482	KF988617	KF988752	Zamora et al. (2014)
*Geastrumcoronatum*	Spain	Zamora 266	KF988361	KF988483	KF988618	KF988753	Zamora et al. (2014)
*Geastrumcoronatum*	Sweden	Zamora 522	KF988362	KF988484	KF988619	KF988754	Zamora et al. (2014)
*Geastrumelegans*	Spain	Herb. Zamora 189	KF988366	KF988488	KF988623	KF988758	Zamora et al. (2014)
*Geastrumelegans*	Sweden	UPS F-560810	KF988367	KF988489	KF988624	KF988759	Zamora et al. (2014)
***Geastrumfibulatum***	**China**	**CLZhao 36066**	**PQ484151**	**PQ481919**	**PQ811828**	–	**Present study**
***Geastrumfibulatum***	**China**	**CLZhao 36067**	**PQ484152**	**PQ481920**	**PQ811829**	–	**Present study**
***Geastrumfibulatum***	**China**	CLZhao 36068*	**PQ484153**	**PQ481921**	**PQ811830**	–	**Present study**
***Geastrumfibulatum***	**China**	**CLZhao 36069**	**PQ484154**	**PQ481922**	**PQ811831**	–	**Present study**
*Geastrumfloriforme*	Spain	MA-Fungi 69173	KF988372	KF988494	KF988629	KF988764	Zamora et al. (2014)
*Geastrumfloriforme*	Spain	Herb.Zamora 453	KF988373	KF988495	KF988630	KF988765	Zamora et al. (2014)
*Geastrumfornicatum*	Spain	MA-Fungi 30749	KF988375	KF988497	KF988632	KF988767	Zamora et al. (2014)
*Geastrumfornicatum*	Spain	Herb.Zamora 255	KF988374	KF988496	KF988631	KF988766	Zamora et al. (2014)
*Geastrumfuscogleba*	USA	NY Trappe 1071	KF988376	KF988498	KF988633	KF988768	Zamora et al. (2014)
*Geastrumfuscogleba*	USA	NY Trappe 9500	KF988377	KF988499	KF988634	KF988769	Zamora et al. (2014)
*Geastrumglaucescens*	Argentina	MA-Fungi 83762	KF988378	KF988500	KF988635	KF988770	Zamora et al. (2014)
*Geastrumglaucescens*	Argentina	MA-Fungi 83763	KF988379	KF988501	KF988636	KF988771	Zamora et al. (2014)
*Geastrumhariotii*	Argentina	MA-Fungi 83765	KF988381	KF988504	KF988639	KF988774	Zamora et al. (2014)
*Geastrumhariotii*	Dominican	MA-Fungi 80070	–	KF988503	KF988638	KF988773	Zamora et al. (2014)
*Geastrumhieronymi*	Argentina	MA-Fungi 83766	KF988384	KF988508	KF988643	KF988776	Zamora et al. (2014)
*Geastrumhieronymi*	Argentina	MA-Fungi 83767	KF988344	KF988509	KF988644	KF988777	Zamora et al. (2014)
*Geastrumhirsutum*	Brazil	UFRN-Fungos 1214	KJ127029	JQ683662	-	JQ683670	Accioly et al. (2019)
*Geastrumhungaricum*	Czech	Sunhede 5993	KP687500	KP687461	KP687542	KP687582	Zamora et al. (2015)
*Geastrumhungaricum*	Spain	Zamora 611	KP687501	KP687462	KP687543	KP687583	Zamora et al. (2015)
*Geastrumjavanicum*	Brazil	UFRN-Fungos 1215	KJ127031	–	–	KJ127016	Cabral et al. (2017)
*Geastrumkotlabae*	Spain	MA-Fungi 39563	KF988385	KF988510	KF988645	KF988778	Zamora et al. (2014)
*Geastrumkotlabae*	Spain	Herb.Zamora 440	KF988386	KF988511	KF988646	KF988779	Zamora et al. (2014)
*Geastrumkuharii*	Argentina	MA-Fungi 83795	KF988463	KF988598	KF988733	KF988864	Zamora et al. (2014)
*Geastrumkuharii*	Argentina	MA:Fungi 86913	KP687502	KP687463	KP687544	KP687584	Zamora et al. (2014)
*Geastrumlaneum*	China	HMJAU65711	OP964640	OP964638	–	–	Wang and Bau (2023)
*Geastrumlaneum*	China	HMJAU65704	OP964641	OP964639	–	–	Wang and Bau (2023)
*Geastrumlitchi*	China	HMJAU65716	OQ360756	OP964619	–	–	Wang and Bau (2023)
*Geastrummarginatum*	Spain	MA-Fungi 32395	KP687505	KP687466	KP687547	KP687587	Zamora et al. (2015)
*Geastrummarginatum*	Spain	MA-Fungi 48129	KP687506	KP687467	KP687548	KP687588	Zamora et al. (2015)
*Geastrummelanocephalum*	Sweden	Herb. Sunhede 7737	KF988396	KF988523	KF988658	KF988790	Zamora et al. (2014)
*Geastrummelanocephalum*	Spain	Herb. Zamora 34	KF988395	KF988522	KF988657	KF988789	Zamora et al. (2014)
*Geastrummelanorhynchum*	China	HMJAU65764	OP964617	OP964614	–	–	Wang and Bau (2023)
*Geastrummelanorhynchum*	China	HMJAU65768	OP964618	OP964615	–	–	Wang and Bau (2023)
*Geastrummeridionale*	Spain	Herb. Zamora 252	KF988412	KF988540	KF988675	KF988808	Zamora et al. (2014)
*Geastrummeridionale*	Spain	Zamora 276	KP687513	KP687475	KP687556	KP687595	Zamora et al. (2015)
*Geastrummichelianum*	Spain	Herb. Zamora 227	KF988398	KF988525	KF988660	KF988792	Zamora et al. (2014)
*Geastrummichelianum*	Argentina	Herb. Ribes 231208-31	–	–	KF988661	KF988793	Zamora et al. (2014)
*Geastrummicrophole*	China	HMJAU65720	OP964636	OP964643	–	–	Wang and Bau (2023)
*Geastrummicrophole*	China	HMJAU65721	OP964637	OP964644	–	–	Wang and Bau (2023)
*Geastrumminimum*	Spain	Herb. Zamora 191	KF988400	KF988528	KF988663	KF988795	Wang and Bau (2023)
*Geastrumminimum*	Sweden	Herb. Sunhede 7746	KF988401	KF988529	KF988664	KF988796	Zamora et al. (2014)
*Geastrummirabile*	Japan	KH-JPN10- 701	JN845107	JN845225	–	JN845349	Wu et al. (2024)
*Geastrummirabile*	Japan	KH-JPN10- 711	JN845108	JN845226	–	JN845350	Wu et al. (2024)
*Geastrummongolicum*	China	HMJAU65762	OP964647	OP964645	–	–	Wang and Bau (2023)
*Geastrummongolicum*	China	HMJAU65763	OP964648	OP964646	–	–	Wang and Bau (2023)
*Geastrumovalisporum*	Bolivia	MA-Fungi 47184	KF988411	KF988539	KF988674	KF988805	Zamora et al. (2015)
*Geastrumoxysepalum*	China	HMJAU65727	OP964632	OP964622	–	–	Wang and Bau (2023)
*Geastrumoxysepalum*	China	HMJAU65728	OP964633	OP964623	–	–	Wang and Bau (2023)
*Geastrumpapinuttii*	Argentina	MA-Fungi 83764	KF988380	KF988502	KF988637	KF988772	Zamora et al. (2015)
*Geastrumpapinuttii*	Argentina	MA-Fungi 86912	KP687515	KP687477	KP687558	KP687596	Zamora et al. (2015)
*Geastrumparvistriatum*	Spain	MA-Fungi 69583	JN943160	JN939560	JN991291	KF988806	Zamora et al. (2014)
*Geastrumparvistriatum*	Spain	Zamora 285	JN943161	JN939571	JN991282	KP687597	Zamora et al. (2015)
*Geastrumpectinatum*	Spain	MA-Fungi 28156	KP687516	KP687478	KP687559	KP687598	Zamora et al. (2015)
*Geastrumpectinatum*	Spain	Zamora 292	KP687521	KP687483	KP687564	KP687601	Zamora et al. (2015)
*Geastrumpleosporum*	Cameroon	MA-Fungi 56971	KF988416	KF988544	KF988679	KF988811	Zamora et al. (2014)
*Geastrumpouzarii*	Czechoslovakia	MA-Fungi 2944	KF988417	KF988545	KF988680	KF988812	Zamora et al. (2014)
*Geastrumpouzarii*	Czechoslovakia	Herb. Sunhede 7494	KF988418	KF988546	KF988681	KF988813	Zamora et al. (2014)
*Geastrumpseudosaccatum*	China	HMJAU65781	OP964625	OP964635	–	–	Wang and Bau (2023)
*Geastrumpseudosaccatum*	China	HMJAU65769	OP964628	OP964634	–	–	Wang and Bau (2023)
*Geastrumquadrifidum*	Spain	Zamora 170	KF988421	KF988549	KF988684	KF988816	Zamora et al. (2014)
*Geastrumquadrifidum*	Spain	Zamora 300	KP687524	KP687486	KP687567	KP687604	Zamora et al. (2015)
*Geastrumrufescens*	Spain	Herb. Zamora 253	KF988424	KF988552	KF988687	KF988819	Zamora et al. (2014)
*Geastrumrufescens*	Spain	Herb. Zamora 274	KF988425	KF988553	KF988688	KF988820	Zamora et al. (2014)
*Geastrumsaccatum*	Spain	Herb.Zamora 260	KF988430	KF988560	KF988695	KF988827	Zamora et al. (2014)
*Geastrumsaccatum*	Spain	Herb.Zamora 461	KF988431	KF988561	KF988696	KF988828	Zamora et al. (2014)
*Geastrumsanglinense*	China	HMSAU 15023	OP050118	OP050163	–	OP056323	Wu et al. (2024)
*Geastrumsanglinense*	China	HMSAU 15024	OP050119	OP050164	–	OP056324	Wu et al. (2024)
*Geastrumsanglinense*	China	HMSAU 15020	OP050116	OP050161	–	–	Wu et al. (2024)
*Geastrumsanglinense*	China	HMSAU 15021	OP050117	OP050162	–	–	Wu et al. (2024)
*Geastrumsanglinense*	China	HMSAU 15025	OP050120	OP050165	–	–	Wu et al. (2024)
*Geastrumschmidelii*	Spain	Herb. Zamora 279	KF988434	KF988564	KF988699	KF988831	Zamora et al. (2014)
*Geastrumschmidelii*	Sweden	UPSF-560805	KF988435	KF988565	KF988700	KF988832	Zamora et al. (2014)
*Geastrumschweinitzii*	Panama	MA-Fungi 36141	KF988438	KF988568	KF988703	KF988835	Zamora et al. (2014)
*Geastrumsetiferum*	Argentina	MA-Fungi 83781	–	KF988571	KF988706	KF988837	Zamora et al. (2014)
*Geastrumsetiferum*	Argentina	MA-Fungi 83782	–	KF988572	KF988707	KF988838	Zamora et al. (2014)
***Geastrumsinense***	**China**	**CLZhao 36029**	**PQ484155**	**PQ481929**	**PQ822016**	–	**Present study**
***Geastrumsinense***	**China**	**CLZhao 36031**	**PQ484156**	**PQ481930**	**PQ822017**	**PQ645133**	**Present study**
***Geastrumsinense***	**China**	**CLZhao 36032**	**PQ484157**	**PQ481923**	**PQ822018**	–	**Present study**
***Geastrumsinense***	**China**	CLZhao 36033*	**PQ484158**	**PQ481924**	**PQ822019**	–	**Present study**
***Geastrumsinense***	**China**	**CLZhao 36038**	**PQ484159**	**PQ481925**	**PQ822020**	**PQ645134**	**Present study**
*Geastrumsmardae*	Canada	Herb. Lebeuf HRL 0160	KF988440	KF988573	KF988708	KF988839	Zamora et al. (2014)
*Geastrumsmardae*	Spain	Herb. Zamora 527	KF988441	KF988574	KF988709	KF988840	Zamora et al. (2014)
*Geastrumstriatum*	Spain	Zamora 242	JN943163	JN939559	JN991290	KP687606	Zamora et al. (2015)
*Geastrumstriatum*	Spain	MA-Fungi 86672	KF988443	KF988577	KF988712	KF988843	Zamora et al. (2015)
*Geastrumsuae*	China	HKAS 123795	ON529511	ON529515	–	–	Zhang et al. (2022)
*Geastrumsuae*	China	HKAS 123796	ON529514	ON529518	–	–	Zhang et al. (2022)
*Geastrumtaylorii*	Argentina	CORDEN9	JN845204	JN845329	–	JN845432	Kasuya et al. (2012)
*Geastrumtenuipes*	Australia	CANB 738350	KP687526	KP687488	KP687570	KP687609	Zamora et al. (2015)
*Geastrumtenuipes*	USA	CANB 775658	KP687527	KP687489	KP687571	KP687610	Zamora et al. (2014)
*Geastrumthanatophilum*	USA	MICH 72012	KF988364	KF988486	KF988621	KF988756	Zamora et al. (2014)
*Geastrumthanatophilum*	USA	MICH 72014	KF988365	KF988487	KF988622	KF988757	Zamora et al. (2014)
***Geastrumtrachelium***	**China**	**CLZhao 35939**	**PQ484160**	**PQ481926**	**PQ783782**	–	**Present study**
***Geastrumtrachelium***	**China**	**CLZhao 36055**	**PQ484162**	**PQ481928**	**PQ783784**	–	**Present study**
***Geastrumtrachelium***	**China**	CLZhao 35992*	**PQ484161**	**PQ481927**	**PQ783783**	–	**Present study**
*Geastrumtriplex*	Madagascar	UPS F-014630-213863	KF988444	KF988578	KF988713	KF988844	Zamora et al. (2014)
*Geastrumtriplex*	Argentina	MA-Fungi 83784	KF988445	KF988579	KF988714	KF988845	Zamora et al. (2014)
*Geastrumvelutinum*	Argentina	MA-Fungi 83785	KF988446	KF988581	KF988716	KF988847	Zamora et al. (2014)
*Geastrumvelutinum*	Argentina	MA-Fungi 83786	KF988447	KF988582	KF988717	KF988848	Zamora et al. (2014)
*Geastrumyanshanense*	China	BJTC 381	MZ508878	MZ509383	MZ571175	MZ571184	Zhou et al. (2022)
*Geastrumyanshanense*	China	BJTC 057	MZ508879	MZ509384	MZ571176	MZ571185	Zhou et al. (2022)
*Geastrumyunnanense*	China	CLZhao 24800	PP511307	–	–	–	Yang et al. (2024)
*Geastrumyunnanense*	China	CLZhao 24893	PP511308	PP511310	–	–	Yang et al. (2024)
*Geastrumyunnanense*	China	CLZhao 24922	PP511309	PP511311	–	–	Yang et al. (2024)
*Schenellapityophila*	Spain	Herb. Zamora 530	KF988346	KF988464	KF988599	KF988734	Zamora et al. (2014)

Sequences generated for this study were aligned, with additional sequences downloaded from GenBank. Sequences were aligned in MAFFT 7 (https://mafft.cbrc.jp/alignment/server/) adjusting the direction of nucleotide sequences according to the first sequence (accurate enough for most cases), and selecting the G-INS-i iterative refinement method (Katoh et al. 2019). The alignment was adjusted manually using AliView version 1.27 (Larsson 2014). The dataset was aligned first, and then the sequences of ITS+nrLSU+RPB1+ATP6 were combined with Mesquite version 3.51. The combined ITS+nrLSU+RPB1+ATP6 sequences were used to infer the position of four new species in the genus *Geastrum* and related species. Sequences of *Schenellapityophila* (Malençon & Riousset) Estrada & Lado retrieved from GenBank were used as an outgroup taxon (Zamora 2015; Wang and Bau 2023).

Maximum Parsimony (MP), Maximum Likelihood (ML) and Bayesian Inference (BI) analyses were applied to the combined three datasets following a previous study (Zhao and Wu 2017). MP analysis was performed in PAUP* version 4.0b10 (Swofford 2002). All characters were equally weighted and gaps were treated as missing data. Trees were inferred using the heuristic search option with TBR branch swapping and 1,000 random sequence additions. Maxtrees were set to 5,000, branches of zero length were collapsed and all parsimonious trees were saved. Clade robustness was assessed using bootstrap (BT) analysis with 1,000 pseudo-replicates (Felsenstein 1985). Descriptive tree statistics - tree length (TL), composite consistency index (CI), composite retention index (RI), composite rescaled consistency index (RC) and composite homoplasy index (HI) were calculated for each maximum parsimonious tree generated. Maximum likelihood (ML) analysis was performed using the CIPRES Science Gateway (Miller et al. 2012) based on the dataset using the RAxML -HPC BlackBox tool, with setting RAxML -HPC BlackBox halt bootstrapping automatically and 0.25 for maximum hours and obtaining the best tree using ML search. Other parameters in ML analysis used default settings, and statistical support values were obtained using nonparametric bootstrapping with 1, 000 replicates.

**Figure 1.**
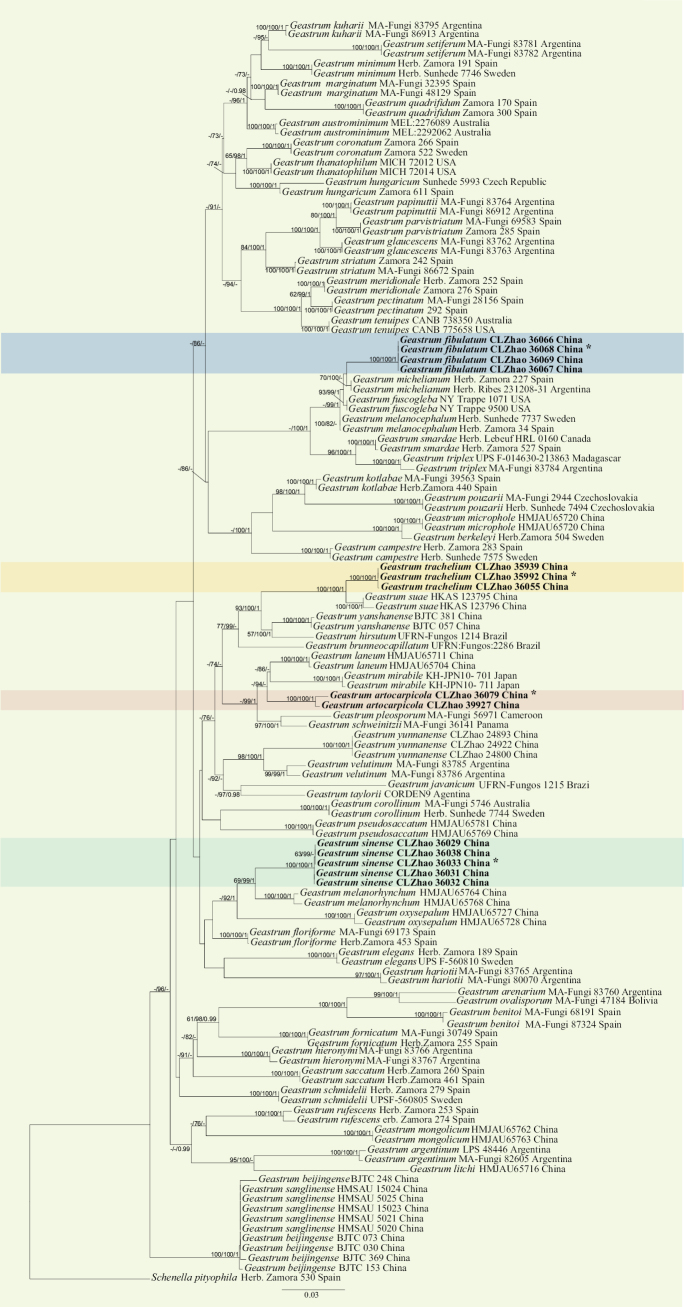
Maximum likelihood strict consensus tree illustrating the phylogeny of four new species and related species in *Geastrum* based on ITS+nrLSU+RPB1+ATP6 sequences. Branches are labeled with maximum likelihood bootstrap values ≥ 70%, parsimony bootstrap values > 50% and Bayesian posterior probabilities ≥ 0.95, respectively. New species accessions are in bold, * type material

The best-evolutionary model of each alignment was estimated using jModelTest (Guindon and Gascuel 2003; Posada 2008) under the Akaike information criterion. MrModeltest 2.3 (Nylander 2004) was used to determine the best-fit evolution model for the dataset for Bayesian Inference (BI). Bayesian Inference was performed with MrBayes 3.1.2 with a general time reversible (GTR+I+G) model of DNA substitution and a gamma distribution rate variation across sites (Ronquist et al. 2012). Four Markov chains were run for two runs from random starting trees for 12 million generations combined ITS+nrLSU+RPB1+ATP6 sequences (Fig. 1), with trees and parameters sampled every 100^th^ generations. The first quarter of all the generations were discarded as burn-ins. A majority rule consensus tree of all remaining trees and posterior probabilities were calculated. Branches were considered significantly supported if they received a maximum likelihood bootstrap value (BS) of ≥ 70%, a maximum parsimony bootstrap value (BT) of ≥ 50%, or Bayesian posterior probabilities (BPP) of ≥ 0.95. The sequence alignments were deposited in figshare (DOI: 10.6084/m9.figshare.28130300).

## ﻿Results

### ﻿Phylogenetic analyses

The combined ITS+nrLSU+RPB1+ATP6 dataset (Fig. 1) included sequences from 128 fungal specimens representing 62 species. The dataset had an aligned length of 3,511 characters, of which 2,389 characters were constant, 193 were variable and parsimony-uninformative and 929 were parsimony-informative. Maximum parsimony analysis yielded one equally parsimonious tree (TL = 5,572, CI = 0.3087, HI = 0.6913, RI = 0.6999 and RC = 0.2161). The best model for the ITS dataset estimated and applied in the Bayesian analysis was GTR+I+G (lset nst = 6; rates = invgamma; prset statefreqpr = dirichlet (1, 1, 1, 1). The bayesian and ML analyses resulted in similar topology to that of the MP analysis with an average standard deviation of split frequencies = 0.009556 (BI), and the effective sample size (ESS) across the two runs is double the average ESS (avg. ESS) = 2491.5. Branches that received bootstrap support for ML and BI ≥ 70%, and 0.95 were considered significantly supported, respectively. The results of BLAST queries in NCBI, based on ITS+nrLSU+RPB1+ATP6 separately, showed the sequences producing significant alignment descriptions.

In ITS BLAST results of *Geastrumartocarpicola*, the top ten taxa were *G.schweinitzii* (Berk. & M.A. Curtis) Zeller, *G.laneum* T. Bau & Xin Wang, *G.pusillipilosum* J.O. Sousa, Alfredo, R.J. Ferreira, M.P. Martín & Baseia, and *G.baculicrystallinum* J.O. Sousa, Accioly, Baseia & M.P. Martín (Maximum record descriptions: Max score 850; Total score 850; Query cover 91%; E value 0; Ident 94.28%). In nrLSU BLAST results, the top ten taxa were *Geastrumflexuosum* (L.S. Domínguez & Castellano) Jeppson & E. Larss., *G.yanshanense*, *G.striatum* DC., *G.lageniforme* Vittad., *G.schweinitzii*, *G.xerophilum* Long ex Desjardin, *G.velutinum* Morgan, and *G.saccatum* Fr. (Maximum record descriptions: Max score 1495; Total score 1495; Query cover 98%; E value 0.0; Ident 98.81%). In RPB1 BLAST results, the top ten taxa were *Geastrumschweinitzii*, *G.pleosporum* Douanla-Meli, *G.yanshanense*, and *G.elegans* Vittad. (Maximum record descriptions: Max score 1256; Total score 1256; Query cover 87%; E value 0.0; Ident 94.82%). In ATP6 BLAST results, the top ten taxa were *Geastrummirabile* Mont., *G.schweinitzii*, and *G.pleosporum* (Maximum record descriptions: Max score 990; Total score 990; Query cover 96%; E value 0.0; Ident 91.73%).

In ITS BLAST results of *Geastrumfibulatum*, the top ten taxa were *G.triplex* Jungh. (Maximum record descriptions: Max score 957; Total score 957; Query cover 92%; E value 0; Ident 96.43%). In nrLSU BLAST results, the top ten taxa were *Geastrummelanocephalum* (Czern.) V.J. Staněk, *G.triplex*, *G.floriforme* Vittad., *G.smardae* V.J. Staněk, *G.flexuosum*, and *G.saccatum* (Maximum record descriptions: Max score 2501; Total score 2501; Query cover 99%; E value 0.0; Ident 99.35%). In RPB1 BLAST results, the top ten taxa were *Geastrummichelianum* (Sacc.) W.G. Sm., *G.melanocephalum*, *G.fuscoglebum* (Zeller) Jeppson & E. Larss., *G.smardae*, and *G.triplex* (Maximum record descriptions: Max score 1982; Total score 1982; Query cover 85%; E value 0.0; Ident 98.74%).

In ITS BLAST results of *Geastrumsinense*, the top ten taxa were *G.saccatum*, *G.melanorhynchum* T. Bau & Xin Wang, *G.fimbriatum* Fr., and *G.lageniforme* Vittad. (Maximum record descriptions: Max score 791; Total score 791; Query cover 96%; E value 0; Ident 90.72%). In nrLSU BLAST results, the top ten taxa were *Geastrumfloriforme*, *G.xerophilum*, *G.saccatum*, *G.fimbriatum*, *G.schmidelii*, *G.striatum*, and *G.gorgonicum* (Maximum record descriptions: Max score 2425; Total score 2425; Query cover 98%; E value 0.0; Ident 98.48%). In RPB1 BLAST results, the top ten taxa were *Geastrumsaccatum*, *G.lageniforme*, *G.corollinum* (Batsch) Hollós, and *G.flexuosum* (Maximum record descriptions: Max score 1487; Total score 1487; Query cover 85%; E value 0.0; Ident 90.30%). In ATP6 BLAST results, the top ten taxa were *Geastrumlageniforme*, and *G.fimbriatum* (Maximum record descriptions: Max score 1035; Total score 1035; Query cover 94%; E value 0.0; Ident 93.30%).

In ITS BLAST results of *Geastrumtrachelium*, the top ten taxa were *G.suae* Z.Q. Zhang, C.H. Li & Z.L. Luo, *G.rubellum* P.-A. Moreau & Lécuru, and *G.triplex* (Maximum record descriptions: Max score 946; Total score 946; Query cover 94%; E value 0; Ident 95.19%). In nrLSU BLAST results, the top ten taxa were *Geastrumyanshanense*, *G.floriforme*, *G.schweinitzii*, and *G.saccatum* (Maximum record descriptions: Max score 2431; Total score 2431; Query cover 99%; E value 0.0; Ident 98.55%). In RPB1 BLAST results, the top ten taxa were *Geastrumyanshanense*, *G.schweinitzii*, and *G.velutinum* (Maximum record descriptions: Max score 1230; Total score 1230; Query cover 97%; E value 0.0; Ident 92.56%).

The topology based on ITS+nrLSU+RPB1+ATP6 sequences, showed that all of the four new taxa were clustered into the genus *Geastrum. G.fibulatum* was sister to *G.michelianum*, *G.sinense* was closely related to *G.melanorhynchum*, *G.trachelium* was sister to *G.suae*, and *G.artocarpicola* formed a monophyletic lineage.

### ﻿Taxonomy

#### ﻿ Geastrum artocarpicola

Taxon classificationFungiGeastralesGeastraceae

X. Yang & C.L. Zhao sp. nov.

B4602CBD-CB52-53D3-B250-B047FF41C50D

856264

Figs 2 , 3

##### Holotype.

China • Guangdong Province, Yangjiang, Jiangcheng District, Bitian Lake Park, 21°52'N, 111°58'E, elev. 27 m, on the living tree of *Artocarpus*, 7 June 2024, CLZhao 36079 (SWFC).

##### Etymology.

*Artocarpicola* (Lat.): referring to the species growing on *Artocarpus*.

##### Description.

**Fruiting body: *Unexpanded basidiomata*** 6–11 mm in diameter, 5–8 mm in height, pyriform to ellipsoidal, lightly pink to pinkish buff when fresh, pinkish buff to gray pink upon drying. ***Expanded basidiomata*** small sized, 0.8–2 cm in diameter, 0.7–1 cm in height. ***Exoperidium***: shallowly saccate to deep saccate, dehiscence often less than halfway or halfway down, splits into 7–8 lobes at maturity, lobes 2–6 mm wide, tapered at the front end, rolled outwards, soft and thin upon drying. ***Mycelial layer*** gray pink, without debris, not easily dislodged. ***Fibrous layer*** white, tightly attached to the mycelial layer. ***Pseudoparenchymatous layer*** smooth surface, flesh-pink, easily exfoliation, dried thin, aseptic collar.

**Figure 2.**
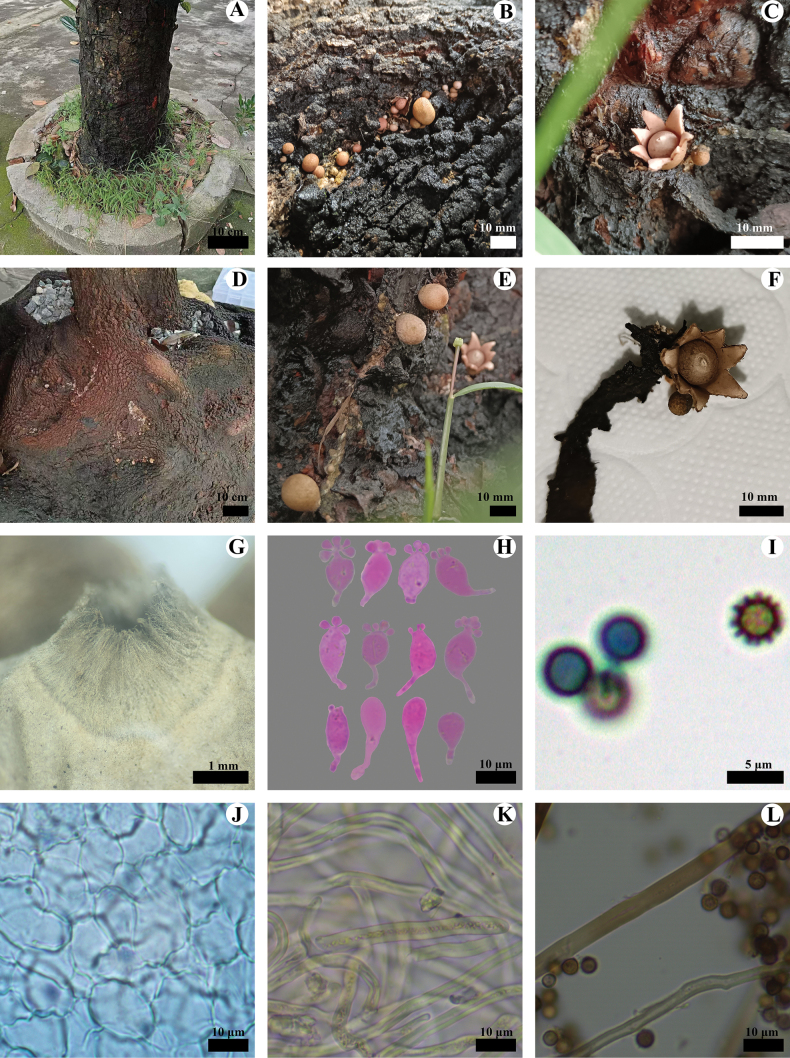
*Geastrumartocarpicola* (CLZhao 36079, CLZhao 39927) **A, D** the environment of *G.artocarpicola***B, E** unexpanded basidiomata **C, F** basidiomata **G** peristome **H** basidia **I** basidiospores **J** pseudoparenchymatous layer **K** mycelium hyphae **L** capillital hyphae.

**Endoperidial body**: Globular, 4–6 mm in diameter, projecting apically, 1–2 mm length, sessile. ***Endoperidium*** slightly pink when fresh, pale mouse-gray upon drying, with a smooth surface under the dissecting microscope. ***Peristome***: wide conical, fibrillose, lighter in color than the endoperidium, distinctly delimited.

**Figure 3.**
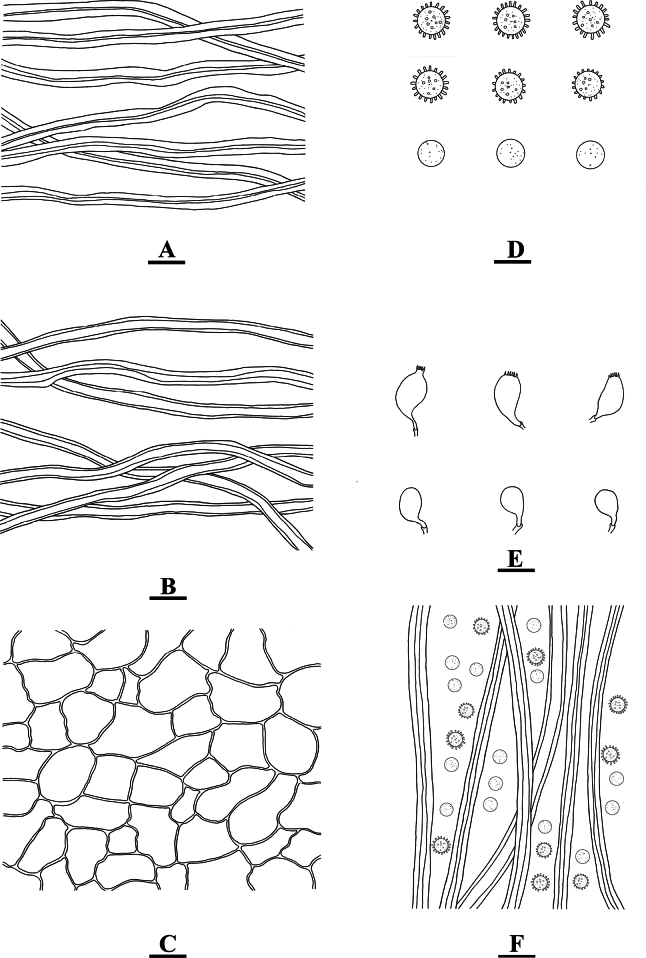
*Geastrumartocarpicola* (CLZhao 36079) **A** mycelium hyphae **B** fibrous hyphae **C** pseudoparenchymatous layer **D** basidiospores **E** basidia **F** capillital hyphae. Scale bars: 10 µm (**A–C, E, F**); 5 µm (**D).**

**Hyphal structure: *Capillitial hyphae***: 2–5.5 µm in diameter, thick-walled, tawny, unbranched, IKI–, CB–, tissues unchanged in KOH. ***Exoperidium mycelium layer*** outside, mycelium hyphae thick-walled, 3–5 µm in diameter; ***fibrous layer*** in the middle, formed of the interlacing filament tissue, fibrous hyphae slightly thick-walled, 3–4.5 µm in diameter; ***pseudoparenchymatous layer*** inside, formed of the angular cell structured, 12–31 × 7–21 µm.

**Basidiospores**: Spherical, (3–)3.5–4(–4.6) × (3.1–)3.5–4(–4.6) µm in diameter, yellowish brown to dark brown, IKI–, CB–; spore surface smooth or ornamentation verrucose, 0.4–0.6(–0.8) µm in length, Q = 1.01, Q_m_ = 1.01 ± 0.01. ***Basidia*** 11–20 × 7–8 μm, bubble-shaped to flask shaped, with 6 inconspicuous basidial pedicels.

##### Additional specimens examined

**(paratypes).** China • Guangdong Province, Yangjiang, Jiangcheng District, Bitian Lake Park, 21°52'N, 111°58'E, elev. 27 m, on living tree of *Dimocarpus*, 30 July 2024, CLZhao 39927 (SWFC).

##### Notes.

Based on ITS+nLSU+RPB1+ATP6 data (Fig. 1), the species *G.artocarpicola* was closely related to both species *G.mirabile* and *G.laneum*. The taxon *G.artocarpicola* resembles *G.laneum* and *G.mirabile* in sharing sessile of endoperidial body. However, *G.laneum* differs from *G.artocarpicola* due to its mycelial layer visible coarse short villus, and its delicately echinulate basidiospores surface (Wang and Bau 2023); *G.mirabile* can be distinguished from *G.artocarpicola* by its expanded basidiomata which has a mycoderm at the base, and capillitial hyphae surface debris (Zhou et al. 2007).

#### ﻿ Geastrum fibulatum

Taxon classificationFungiGeastralesGeastraceae

X. Yang & C.L. Zhao sp. nov.

7DEB6B7D-B9B9-5CEB-9ABD-D1A319C31A75

856265

Figs 4 , 5

##### Holotype.

China • Jiangsu Province, Nanjing, Jiangning County, Diaoyutai Mountain, 31°58'N, 118°55'E, elev. 93.1 m, on the ground, 28 May 2024, CLZhao 36068 (SWFC).

##### Etymology.

*Fibulatum* (Lat.): referring to the generative hyphae with clamp connections of type specimen.

##### Description.

**Fruiting body: *Expanded basidiomata*** small to medium-sized, 4–6 cm in diameter, 1–2.5 cm in height. ***Exoperidium***: shallowly saccate to deep saccate, dehiscence often greater than halfway, splits into 5–6 lobes at maturity, lobes 8–18 mm wide, lobes long and mostly rolled outward, toughened and thin upon drying. ***Mycelial layer*** clay buff to grayish brown when dry, without debris, not easily dislodged. ***Fibrous layer*** white, tightly attached to the pseudoparenchymatous layer. ***Pseudoparenchymatous layer*** smooth surface, white to cream when fresh, cinnamon-buff to olivaceous to grayish brown to fuscous when dry, not deciduous, aseptic collar, thin when dry.

**Figure 4.**
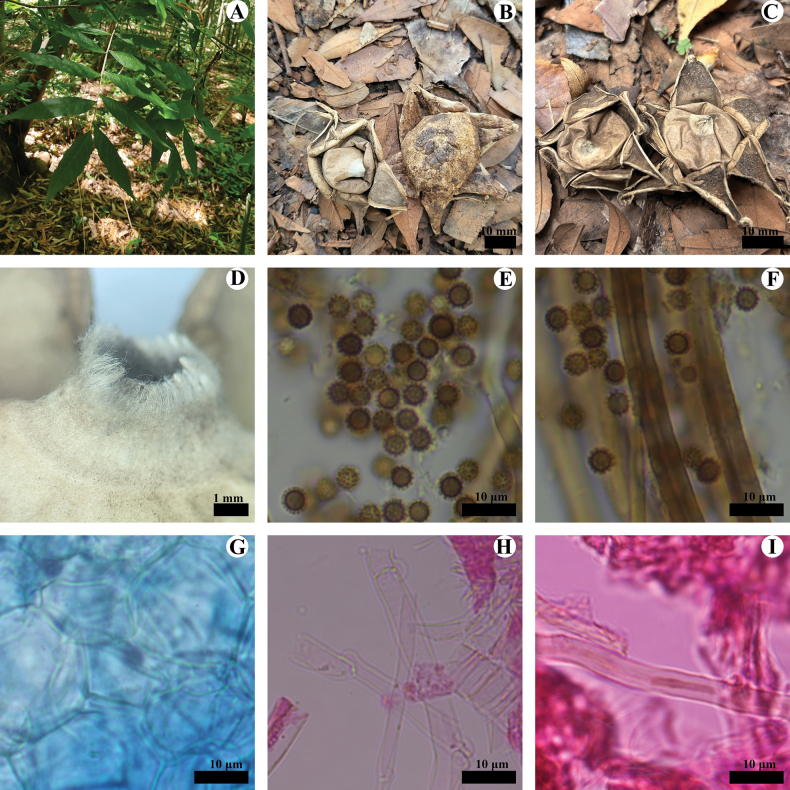
*Geastrumfibulatum* (CLZhao 36066, CLZhao 36067, CLZhao 36068, CLZhao 36069) **A** the environment of *G.fibulatum***B, C** basidiomata **D** peristome **E** basidiospores **F** capillital hyphae **G** pseudoparenchymatous layer **H, I** mycelium hyphae

**Endoperidial body**: Globular, 15–25 mm in diameter, projecting apically, 1–5 mm length, sessile. ***Endoperidium*** of clay pink to clay buff, with a smooth surface and grayish villus visible under the dissecting microscope. ***Peristome*** silky fibrillose, color lighter than the endoperidium, distinctly delimited.

**Figure 5.**
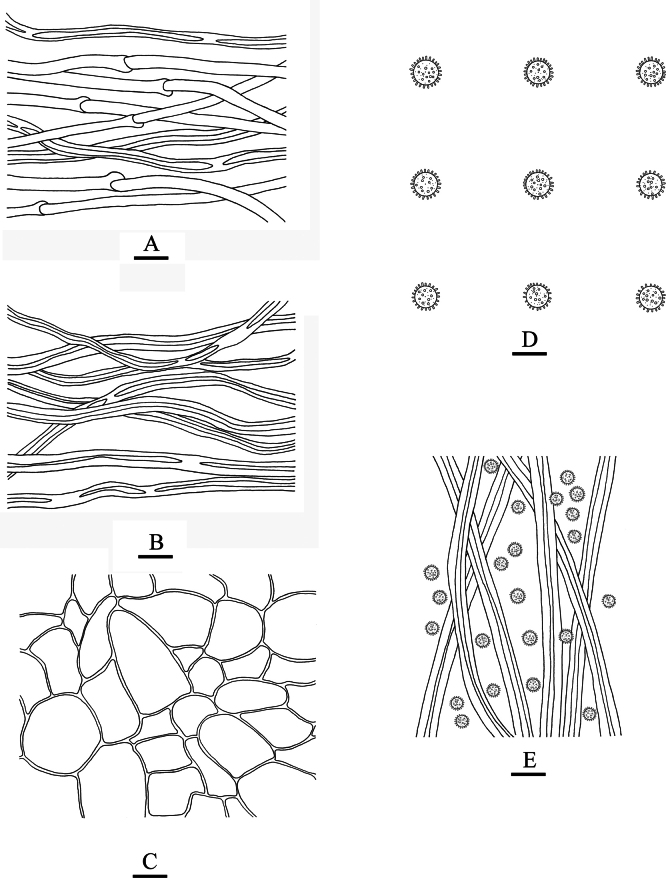
*Geastrumfibulatum* (CLZhao 36068) **A** mycelium hyphae **B** fibrous hyphae **C** pseudoparenchymatous layer **D** basidiospores **E** capillital hyphae. Scale bars: 10 µm (**A–C, E**); 5 µm (**D).**

**Hyphal structure: *Capillitial hyphae*** 3–6 µm in diameter, thick-walled, olivaceous buff, unbranched, IKI–, CB–; tissues unchanged in KOH. ***Exoperidium mycelium layer*** outside, dimitic hyphal system; generative hyphae with clamp connections, thin-walled, colorless, 3.5–6 µm in diameter, skeletal hyphae thick-walled to subsolid, slightly yellowish, 3–5.5 µm in diameter; ***fibrous layer*** in the middle, formed of the interlacing filament tissue, fibrous hyphae thick-walled to solid, 2.5–6 µm in diameter; ***pseudoparenchymatous layer*** inside, formed of the angular cell structured, 18–54 × 10–26 µm.

**Basidiospores**: Spherical, 3–3.6(–4) × 3–3.5(–4) µm in diameter, brown, thick-walled, to grayish brown, IKI–, CB–; spore surface with ornamentation verrucose, 0.5–0.8(–1) µm in length, Q = 1.01, Q_m_ = 1.01 ± 0.01.

##### Additional specimens examined

**(paratypes).** China • Jiangsu Province, Nanjing, Jiangning County, Diaoyutai Mountain, 31°58'N, 118°55'E, elev. 93.1 m, on the ground, 28 May 2024, CLZhao 36066; CLZhao 36067; CLZhao 36069(SWFC).

##### Notes.

Based on dataset of ITS+nLSU+RPB1+ATP6 data (Fig. 1), *Geastrumfibulatum* was sister to *G.michelianum.* The taxon *G.fibulatum* resembles *G.fuscogleba* and *G.melanocephalum* in sharing verrucose basidiospores. However, *G.fuscogleba* differs from *G.fibulatum* by its arched expanded basidiomata, and its larger basidiospores (4.5–7 µm, Domínguez and Castellano 1996); the species *G.melanocephalum* can be distinguished from *G.fibulatum* by its endoperidial body with stalk, and arched expanded basidiomata (Jeppson et al. 2013).

#### ﻿ Geastrum sinense

Taxon classificationFungiGeastralesGeastraceae

X. Yang & C.L. Zhao sp. nov.

BEC2C0C3-D975-579E-A2D3-5C9B33411D6A

856266

Figs 6 , 7

##### Holotype.

China • Fujian Province, Xiamen, Siming District, Xiamen Botanical Garden, 24°27'N, 118°6'E, elev. 179 m, on the ground, 24 May 2024, CLZhao 36033 (SWFC).

##### Etymology.

*Sinense* (Lat.): referring to the species being found in China.

##### Description.

**Fruiting body: *Expanded basidiomata*** medium sized, 10–25 mm in height, 15–40 mm in diameter. ***Exoperidium***: arched, dehiscence often halfway down, splits into 5–9 lobes at maturity, lobes 3–10 mm wide, mostly rolled outward to under the outer exoperidial disc, tapered at the front end, turn to soft and thin upon drying. ***Mycelial layer***: pinkish buff, without debris, not easily dislodged. ***Fibrous layer***: white, tightly attached to the mycelial layer. ***Pseudoparenchymatous layer***: smooth surface, cream to pink, not deciduous, aseptic collar when fresh, clay-buff to grayish brown, thinner and soft when dry.

**Figure 6.**
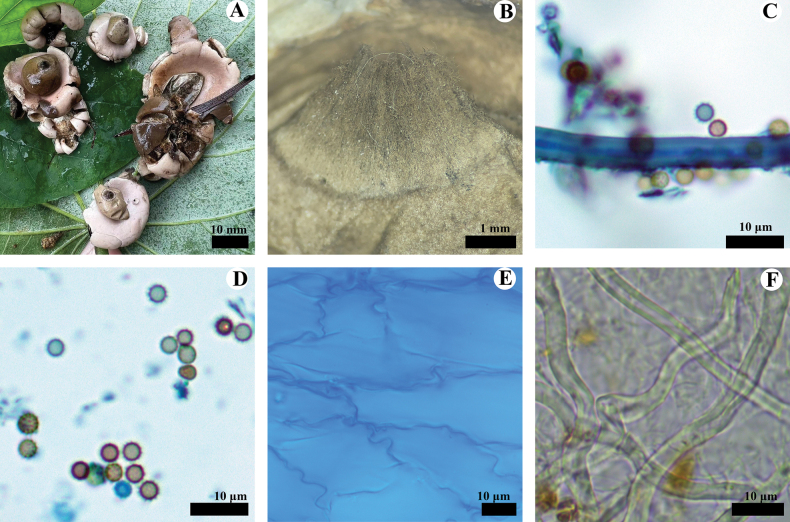
*Geastrumsinense* (CLZhao 36029, CLZhao 36031, CLZhao 36032, CLZhao 36033, CLZhao 36038) **A** basidiomata **B** peristome **C** capillital hyphae **D** basidiospores **E** pseudoparenchymatous layer **F** mycelium hyphae.

**Endoperidial body**: Globular, 8–15 mm in diameter, projecting apically, 1–3 mm length, sessile. ***Endoperidium*** pale to dark brown. ***Peristome*** broadly conical, fibrillose, dark brown to gray, distinctly delimited.

**Hyphal structure: *Capillitial hyphae***: up to 4.5–6.5 µm in diameter, thick-walled to subsolid, brown, occasionally branched, IKI–, CB–; tissues unchanged in KOH. ***Exoperidium mycelium layer*** outside, mycelium hyphae slightly thick-walled to thick-walled, 4–5 µm in diameter. ***fibrous layer*** in the middle, formed of the interlacing filament tissue, fibrous hyphae slightly thick-walled, 3–4.5 µm in diameter; ***pseudoparenchymatous layer*** inside, formed of the angular cell structured, 20–75 × 13–25 µm;

**Figure 7.**
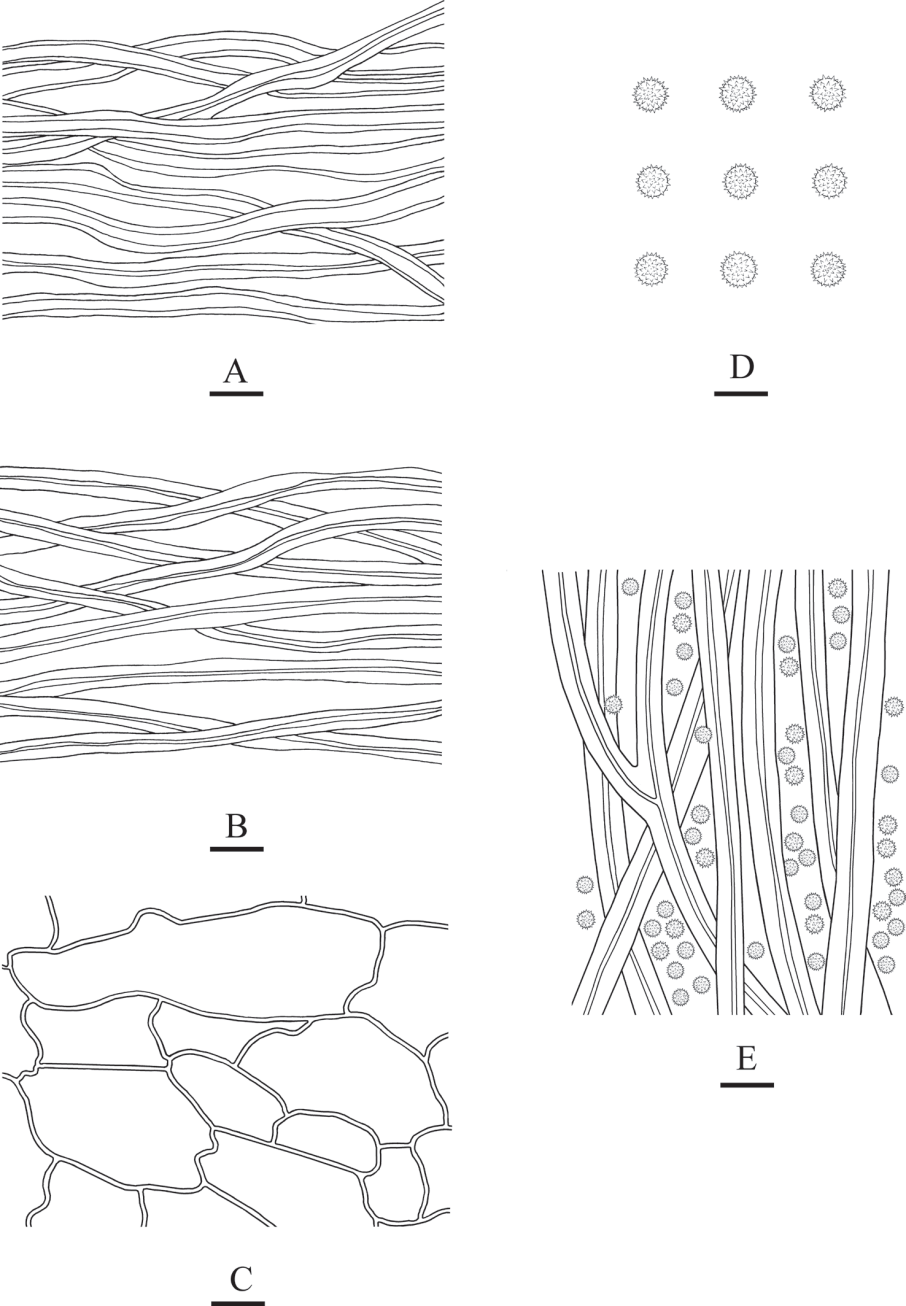
*Geastrumsinense* (CLZhao 36033) **A** mycelium hyphae **B** fibrous hyphae **C** pseudoparenchymatous layer **D** basidiospores **E** capillital hyphae. Scale bars: 10 µm (**A–C, E**); 5 µm (**D).**

**Basidiospores**: Spherical, 2.6–3(–3.5) × 2.5–3(–3.5) µm in diameter, yellowish brown to dark brown, IKI–, CB–; spore surface with echinulate, 0.4–0.8 µm in length, Q = 1.01, Q_m_ = 1.01 ± 0.01.

##### Additional specimens examined

**(paratypes).** China • Fujian Province, Xiamen, Siming, Xiamen Botanical Garden, 24°27'N, 118°6'E, elev. 179 m, on the ground, 24 May 2024, CLZhao 36029; CLZhao 36031; CLZhao 36032 and CLZhao 36038 (SWFC).

##### Notes.

Based on ITS+nLSU+RPB1+ATP6 data (Fig. 1), *G.sinense* was sister to *G.melanorhynchum*. The *G.sinense* resembles *G.oxysepalum* and *G.floriforme* in sharing sessile endoperidial body. However, *G.oxysepalum* differs from *G.sinense* by its shallowly saccate expanded basidiomata, and non-constant peristomal ring (Wang and Bau 2023); *G.floriforme* can be distinguished from *G.sinense* by its deep saccate expanded basidiomata, and larger basidiospores (5.5–7 µm, Zhou et al. 2007)

#### ﻿ Geastrum trachelium

Taxon classificationFungiGeastralesGeastraceae

X. Yang & C.L. Zhao sp. nov.

F10B753D-4F07-5382-B51E-31425720B618

856267

Figs 8 , 9

##### Holotype.

China • Guangdong Province, Guangzhou, Huangpu District, Shuangchuanshi Mts., 23°11'N, 113°32′E, elev. 69.6 m, on the fallen angiosperm leaves, 14 May 2024, CLZhao 35992 (SWFC).

##### Etymology.

*Trachelium* (Lat.): referring to the species having a long stipe.

##### Description.

**Fruiting body: *Unexpanded basidiomata***, 9–12 mm in diameter, ellipsoidal to fusiform, white to pink. ***Expanded basidiomata*** small to medium sized, 2–3 cm in diameter, 1.5–2 cm in height, and long stipe (height 0.5–1 cm). ***Exoperidium***: deep saccate, dehiscence often halfway down, splits into 5–6 lobes at maturity, lobes 5–10 mm wide, tapered at the front end, exoperidium attached to the rhizomorphs, soft and thin upon drying. ***Mycelial layer***: cream to clay-buff when fresh, without debris, not easily dislodged, turning to clay-buff when dry. ***Fibrous layer***: white, tightly attached to the mycelial layer. ***Pseudoparenchymatous layer***: smooth surface, cream to flesh pink when fresh, not deciduous, aseptic collar, turning to gray brown, thinner when dry.

**Figure 8.**
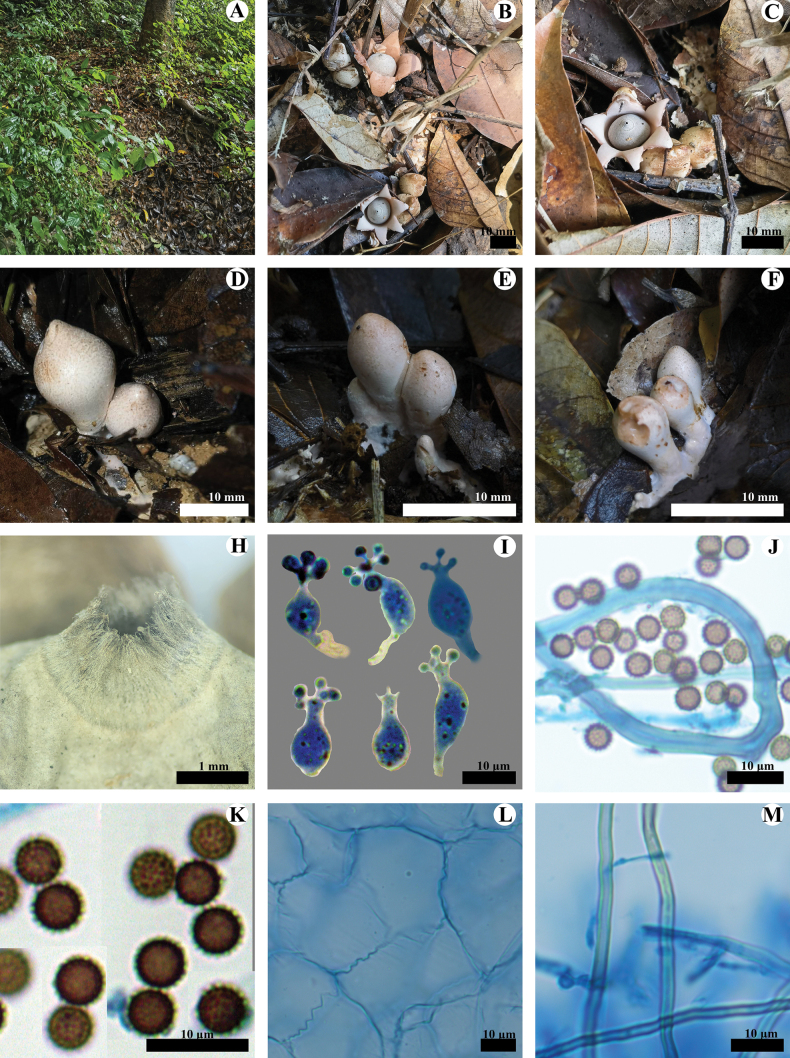
*Geastrumtrachelium* (CLZhao 35939, CLZhao 35992, CLZhao 36055) **A** the environment of *G.trachelium***B, C** basidiomata **D, E, F** unexpanded basidiomata **G** peristome **H** basidia **I** capillital hyphae **J** basidiospores **K** pseudoparenchymatous layer **L** mycelium hyphae.

**Endoperidial body**: Globular, 10–13 mm in diameter, projecting apically, 1–3 mm length, sessile. ***Endoperidium*** ash-gray, with a smooth surface and buff villus visible under the dissecting microscope. ***Peristome*** wide conical, fibrillose, ash-gray to dark gray, distinctly delimited.

**Figure 9.**
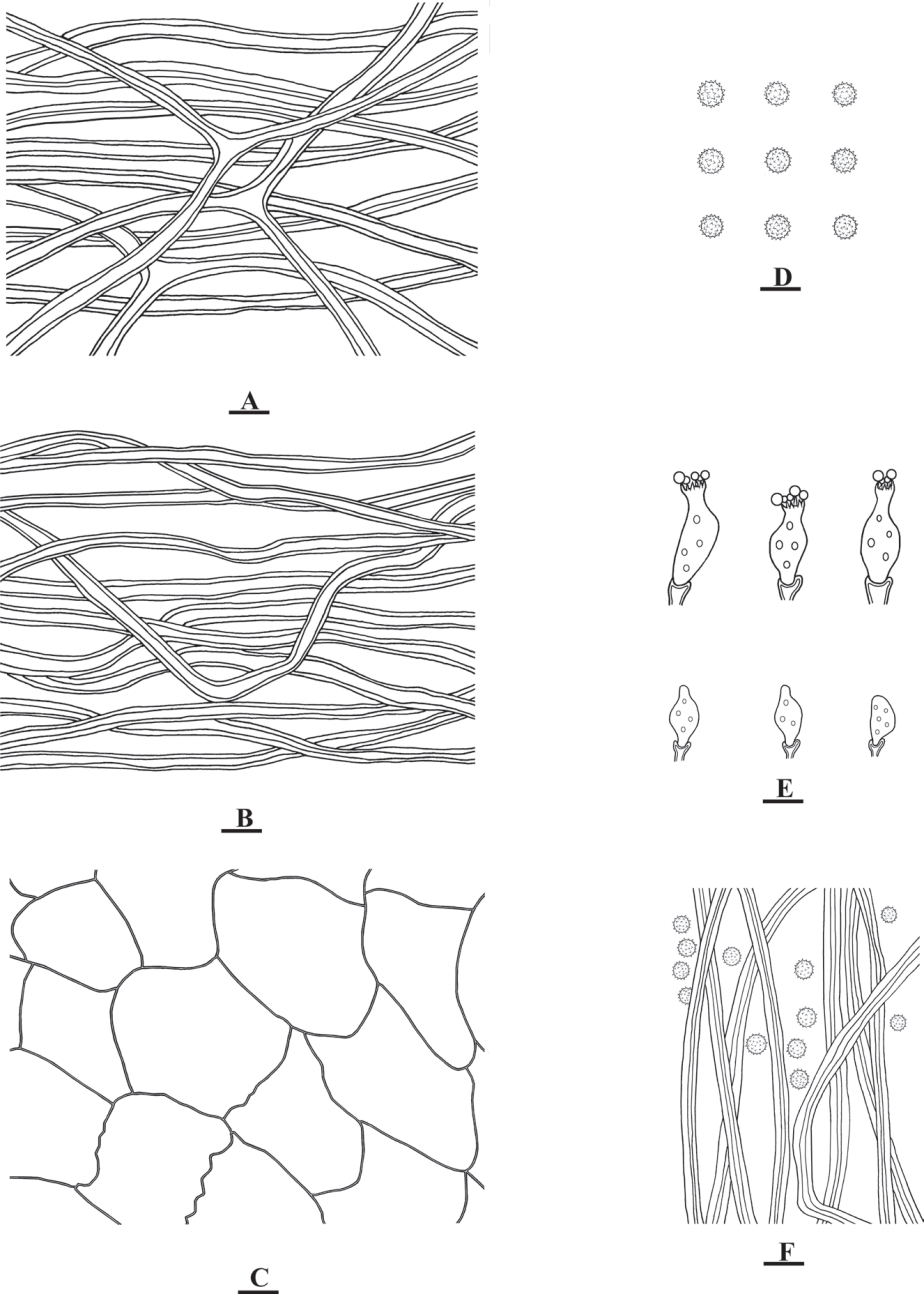
*Geastrumtrachelium* (CLZhao 35992) **A** mycelium hyphae **B** fibrous hyphae **C** pseudoparenchymatous layer **D** basidiospores **E** basidia **F** capillital hyphae. Scale bars: 10 µm (**A–C, E, F**); 5 µm (**D**).

**Hyphal structure: *Capillitial hyphae***: 3–4.5 µm in diameter, thick-walled, slightly yellowish, unbranched, IKI–, CB–; tissues unchanged in KOH. ***Exoperidium mycelium layer*** outside, mycelium hyphae slightly thick-walled to solid, 2.2–3.1 µm in diameter; ***fibrous layer*** in the middle, formed of the interlacing filament tissue, fibrous hyphae slightly thick to thick-walled, 2.5–3.3 µm in diameter; ***pseudoparenchymatous layer*** inside, formed of the angular cell structured, 22–56 × 14.5–33 µm.

**Basidiospores**: Spherical, (3.5–)3.7–4.3(–4.6) × (3.5–)3.8–4.3(–4.6) µm in diameter, grayish brown to dark brown, IKI–, CB–; spore surface with delicately echinulate, 0.4–0.9 µm in length, Q = 1.01, Q_m_ = 1.01 ± 0.01. ***Basidia*** flask shaped, 13.5–18.5 × 6–9 μm, with 6 inconspicuous basidial pedicels.

##### Additional specimens examined

**(paratypes).** China • Guangdong Province, Guangzhou, Huangpu, Shuangchuanshi Mts., 23°11'N, 113°32'E, elev. 69.6 m, on the fallen angiosperm leaves, 9 May 2024, CLZhao 35939; 1 June 2024, CLZhao 36055(SWFC).

##### Notes.

Based on ITS+nLSU+RPB1+ATP6 data (Fig. 1), *G.trachelium* was sister to *G.suae*. The species *G.trachelium* resembles *G.yanshanense* and *G.hirsutum* in sharing sessile endoperidial body. However, *G.yanshanense* differs from *G.trachelium* by its smaller basidiospores (2.7–3.2 × 2.8–3.3 µm, Zhou et al. 2022); *G.hirsutum* can be distinguished from *G.trachelium* by its subglobose to obovate unexpanded basidiomata, and smaller basidiospores (2.5–3 μm, Baseia and Calonge 2006).

#### ﻿ Geastrum beijingense

Taxon classificationFungiGeastralesGeastraceae

C.L Hou, Hao Zhou & Ji Qi Li, Mycosystema 41: 11 (2022)

0526C977-A9FE-514C-B61E-091E966E50F4

570850

##### Typus.

China, Beijing, Yanqing District, Zhangjiaying Village, BJTC 248 (holotype).

#### ﻿ Geastrum sanglinense

Taxon classificationFungiGeastralesGeastraceae

Y.Q. Wu & Shu R. Wang, Mycoscience 65: 13 (2024). Synonym

ADABE0B8-38E5-577D-8BCB-80F114550C5B

845733

##### Typus.

China, Shanxi Province, Yangcheng County, the Manghe Rhesus Monkey National Nature Reserve, located near Huiquan Village, HMSAU 15020 (holotype)

##### Note.

Our results show that the types of *Geastrumbeijingense* and *G.sanglinense* represent a single species, because *G.beijingense* has the priority (Zhou et al. 2022), and *G.sanglinense* is treated as synonym of *beijingense*. In addition, the type localities of these two taxa are very close with similar forest and climate. In the phylogenetic tree based on ITS+LSU+rpb1+atp6 (Fig. 1), both *G.beijingense* and *G.sanglinense* are nested in a lineage.

## ﻿Discussion

In the present study, four new species *Geastrumartocarpicola*, *G.fibulatum*, *G.sinense* and *G.trachelium* are described, based on the phylogenetic analyses and morphological characteristics.

The integrative taxonomy inferred from the ITS and LSU nrDNA, RPB1, and ATP6 revealed an unexpected diversity in the genus *Geastrum* (Geastrales, Basidiomycota). A total of 27 lineages was proposed and a revised taxonomy of the combination of the different sources of taxonomic information was presented (Zamora et al. 2015). Phylogenetically, four new species were nested in the genera *Geastrum* within the order Geastrales, in which *G.fibulatum* was sister to *G.michelianum*, and the *G.sinense* was closely related to *G.melanorhynchum*, and the *G.trachelium* was sister to *G.suae*, and the *G.artocarpicola* formed a monophyletic lineage. However, morphologically, *G.michelianum* differs from *G.fibulatum* by having the arched of the fruitbody, and the collar pseudoparenchymatous layer at the base of endoperideal body (Muñoz Sánche 2020). The species *G.melanorhynchum* differs from *G.sinense* by having the shallowly saccate fruiting body, and larger basidiospores (3.5–3.9 µm) (Wang and Bau 2023). The taxon *G.suae* differs from *G.trachelium* by having larger unexpanded basidiomata (13–28 mm), and high expanded basidiomata (35–70 mm) in, and larger basidiospores (4.5–6 × 5–6 µm; Zhang et al. 2023b).

Morphologically, our new species resemble 16 similar species in the *Geastrum*, individually as *G.fuscoglebum* (Zeller) Jeppson & E. Larss., *G.michelianum*, *G.melanocephalum*, *G.smardae*, *G.triplex*, *G.melanorhynchum*, *G.oxysepalum* T. Bau & Xin Wang, *G.floriforme*, *G.suae*, *G.yanshanense*, *G.hirsutum* Baseia & Calonge, *G.brunneocapillatum* J.O. Sousa, Accioly, M.P. Martín & Baseia, *G.laneum*, *G.mirabile*, *G.pleosporum* and *G.schweinitzii*. A morphological comparison among our new species and sixteen similar species are presented in Table 2.

**Table 2.** A morphological comparison among four new *Geastrum* species and other similar species.

Species name	Unexpanded basidiomata	Expanded basidiomata	Endoperidial body	Basida	Basidiospores	References
*Geastrumartocarpicola*	5–8 mm high, 6–11 mm diam; pyriform to ellipsoidal	Shallowly saccate to deep saccate; 7–10 mm high, 8–20 mm diam; 7–8 rayes	Sessile; 4–6 mm diam; obvious oral margin ring	bubble-shaped to flask shaped; 11–20 × 7–8 μm.	Spherical; 3.5–4 × 3.5–4 μm diam	Present study
*Geastrumbrunneocapillatum*	6–10 mm high, 7–13 mm diam; obpyriform to oval	Sacate; 4.1–11 mm high, 8–26 mm diam; 5–7 rayes	Sessile; 15–25 mm diam; obvious oral margin ring	Thin-walled; clavate; pyriform to lageniform; 12.2–19.3 × 3.8–6.7 μm.	Globose to subglobose; 2.8–4 × 2.7–4 μm diam	Accioly et al. (2019)
*Geastrumfibulatum*	—	Shallowly saccate to deep saccate; 10–25 mm high, 40–60 mm diam; 5–6 rayes	Sessile; 15–25 mm diam; obvious oral margin ring	—	Spherical; 3–3.6 × 3–3.5 μm diam	Present study
*Geastrumfloriforme*	6–10 mm diam; subglobose	Deep saccate; 6–14 mm diam; 6–11 rayes	Sessile; 4–11 mm diam; non-constant peristomal ring	—	Globose to subglobose; 5.5–7 μm diam	Zhou et al. (2007)
*Geastrumfuscogleba*	10–50 mm diam; globose to subglobose	3–4 rayes	12–20 mm diam	Lecythiform to lageniform; 6 sporde; 11 × 7 µm	Globose; 4.5–7 µm diam	Domínguez and Castellano (1996)
*Geastrumhirsutum*	5–10 mm high, 4–8 mm diam; subglobose to obovate	Saccate; 15–20 mm diam when open; 5–7 rayes	Sessile; 4–6 mm diam; obvious oral margin ring	—	Globose; 2.5–3 μm diam	Baseia and Calonge (2006)
*Geastrumlaneum*	3–10 mm in size	Shallowly saccate; 4.5–9.5 mm high; 5–7 rayes	Sessile; 2–7 mm diam	—	Spherical; 2.5–3.9 µm diam	Wang and Bau (2023)
*Geastrummelanocephalum*	—	Arched; 40–200 mm diam; 5–8 rayes	Stalk; 25–60 mm diam; peristome lacking	—	Globose; 3.5–4.5 µm diam	Jeppson et al. (2013)
*Geastrummelanorhynchum*	—	Shallowly saccate, arched; 12–35 mm diam; 7–9 rayes	Sessile; 6–20 mm diam; obvious oral margin ring	Clavate, sublageniform; 4 sporde; 14.4–19.7 × 9.1–11.4 µm	Spherical; 3.5–3.9 µm diam	Wang and Bau (2023)
*Geastrummichelianum*	—	Arched; up to 120 mm diam; 5–6 rayes	Up to 40 mm diam; obvious oral margin ring	—	Globose; up to 6 µm diam	Muñoz (2020)
*Geastrummirabile*	5–10 mm high, 4–8 mm diam; globose, subglobose, obovate	Shallowly saccate to deep saccate; 6–20 mm in high; 5–7 rayes	Sessile; 3–9 mm diam	—	Subglobose to obovate; 2.5–4 µm diam	Zhou et al. (2007)
*Geastrumoxysepalum*	13–16 mm diam	shallowly saccate; 12–17 mm diam; 5–8 rayes	Sessile; 6–13 mm diam; non-constant peristomal ring	—	Spherical; 2.7–3.9 µm diam	Wang and Bau (2023)
*Geastrumpleosporum*	12–15 mm diam; globose to depressed globose	5–6 rayes	Sessile; 5–8 mm diam	Clavate, ventricose to flask-shaped with a more or less long collar; 4–8 spored; 15.5–19 × 4–5.5 µm	Globose, cylindrical, elliptic, reniform, club-shaped; 4–6 × 4–5 µm	Douanla-Meli et al. (2005)
*Geastrumschweinitzii*	oval	Deep saccate; 7–9 mm high × 12–20 mm diam; 5–8 rayes	Sessile; 3–9 mm diam; spherical or oblate spherical; obvious oral margin ring	—	Spherical; 2–3.6 × 2–3.4 µm	Han and Bau (2015)
*Geastrumsinense*	—	Arched; 10–25 mm high, 15–40 mm diam; 5–9 rayes	Sessile; 8–15 mm diam; obvious oral margin ring	—	Spherical; 2.6–3 × 2.5–3 µm	Present study
*Geastrumsmardae*	—	Pseudofornicate; 40–60 mm diam; 7–9 rayes	Shortly stalked; 15–20 mm diam; obvious oral margin ring	—	Globose; 3–4.5 µm diam	Jeppson et al. (2013)
*Geastrumsuae*	13–28 mm high; cylindrical to ellipsoidal	Deep saccate; 35–70 mm high, 18–37 mm diam; 6 rayes; exoperidium attached to the rhizomorphs	Sessile; 11–23 mm high; obvious oral margin ring	—	Globose; 4.5–6 × 5–6 µm	Zhang et al. (2022)
*Geastrumtrachelium*	9–12 mm diam; ellipsoidal to fusiform	Deep saccate; 15–20 mm high, 20–30 mm diam; 5–6 rayes; exoperidium attached to the rhizomorphs	Sessile; 10–13 mm diam; obvious oral margin ring	Flask shaped; 13.5–18.5 × 6–9 μm; 6 sporde	Spherical; 3.7–4.3 × 3.8–4.3 µm	Present study
*Geastrumtriplex*	14–22 mm diam and 15–25 mm high; onion shaped to obpyriform	Saccate; 35–50 mm diam; 5–7 rayes	Sessile; 10–18 mm diam; obvious oral margin ring	—	Globose; 3.6–4.2 × 3.3–4.1 μm diam	Vishal et al. (2023)
*Geastrumyanshanense*	9– 15 mm high; spherical to ellipsoidal	Deep saccate; 15–25 mm high; 5–7 rayes; exoperidium attached to the rhizomorphs	Sessile; 8–13 mm high; obvious oral margin ring	Bubble-shaped to flask shaped; 8.1–9 × 12.6–13.5 μm; with 4–7 sporde	Globose to ellipsoid; 2.7–3.2 × 2.8–3.3 µm	Zhou et al. (2021)

Macrofungi are an important part of forest ecosystems, which are mainly composed of most members of Basidiomycota and some members of Ascomycota, and they possess important economic value and ecological functions (Wu et al. 2019, 2022; Dai et al. 2021; Deng and Zhao 2023; Guan et al. 2023; Yuan et al. 2023b; Zhang et al. 2023a, 2024; Dong et al. 2024a, b; Luo et al. 2024). The family Geastraceae is an extensively studied group of Basidiomycota (Zhou et al. 2007; Finy et al. 2021; Wang and Bau 2023). However, the *Geastrum* species diversity in China is still not well known, especially in the subtropical and tropical areas. Therefore, a multidisciplinary approach, combining taxonomists, molecular biologists and field practitioners, is required. Edible and medicinal fungi have huge potential as food and medicines, especially in Asia and their prospects (Dai and Yang 2008; Dai et al. 2010; Cui et al. 2023; Li et al. 2023; Xie et al. 2024). In the present study, four new species are introduced from China, which will further enrich our knowledge of the macrofungal diversity. We anticipate that more undescribed *Geastrum* taxa will be discovered throughout China after extensive collection combined with morphological and molecular analyses.

## Supplementary Material

XML Treatment for Geastrum artocarpicola

XML Treatment for Geastrum fibulatum

XML Treatment for Geastrum sinense

XML Treatment for Geastrum trachelium

XML Treatment for Geastrum beijingense

XML Treatment for Geastrum sanglinense

## ﻿Additional information

### Author ORCIDs

Xin Yang https://orcid.org/0009-0009-7755-0581

Yonggao Zhu https://orcid.org/0009-0008-5341-3798

Songjing Duan https://orcid.org/0009-0005-3122-0318

Xingxing Wu https://orcid.org/0009-0001-1497-8937

Changlin Zhao https://orcid.org/0000-0002-8668-1075

### Data availability

All of the data that support the findings of this study are available in the main text.
